# Supplementary material for: Diagnostic role of Sysmex hematology analyzer in the detection of malaria: A systematic review and meta-analysis
Source: PLoS One. 2024 Sep 6;19(9):e0296766. doi: 10.1371/journal.pone.0296766 (PMC11379208; doi:10.1371/journal.pone.0296766)
Supplement: Attachment — Submitted filename: PONE-D-23-30250_reviewer AA edits.pdf [file pone.0296766.s002.pdf]

PLOS ONE

Diagnostic role of Sysmex hematology analyzer in the detection of malaria: A systematic review and meta-analysis

--Manuscript Draft--

**Manuscript Number:**	PONE-D-23-30250
**Article Type:**	Research Article
**Full Title:**	Diagnostic role of Sysmex hematology analyzer in the detection of malaria: A systematic review and meta-analysis
**Short Title:**	Diagnostic role of Sysmex hematology analyzer in the detection of malaria
**Corresponding Author:**	Zewudu Mulatie<br>Wollo University<br>Dessie, ETHIOPIA
**Keywords:**	Diagnostic test accuracy, malaria, plasmodium species, Sysmex
**Abstract:**	Background: Malaria control depends primarily on rapid accurate diagnosis followed by successful treatment. Light microscopy is still the "gold standard" approach for diagnosing malaria. The diagnostic test procedure must be accurate enough to detect malaria parasites. This review aimed to investigate the diagnostic accuracy of Sysmex for malaria diagnosis.<br>Methods: From April to June 14, 2023, PubMed, PubMed Central, Science Direct databases, Google Scholar, and Scopus were searched for papers evaluating the diagnostic accuracy of sysmex methods for malaria diagnosis. The studies' methodological quality was assessed using the Quality Assessment of Diagnostic Accuracy Studies -2 tool. Using Review Manager 5.4.1, the estimates of sensitivity and specificity, as well as their 95% confidence intervals, were shown in forest plots. The summary estimates of sensitivity, specificity, positive likelihood ratio, negative likelihood ratio, and diagnostic odds ratio were calculated using Midas in Stata14.0. Diagnostic accuracy testing is expected to be very variable. When the heterogeneity was considerable or the I2 was greater than 50%, the Meta-disc 1.4.0 software was used to determine whether a threshold effect existed.<br>Results: A total of 41 studies published between 2001 and 2020 were examined using review manager, Midas (Stata), and Meta-disc. The sensitivity and specificity of Sysmex for studies ranged from 46% to 100% and 81% to 100%, respectively. The sensitivity and specificity of Sysmex were estimated to be 95% (95% CI: 85%-99%) and 99% (95% CI: 97%-100%), respectively. The area under the curve of Sysmex was 1.00 (95% CI: 0.99-1.00), indicating that the test showed excellent diagnostic accuracy. There was significant heterogeneity among the studies included in this meta-analysis. The summary estimate of sensitivity and specificity of Sysmex using PCR as the gold standard was 97.6% (95% CI: 83.2, 99.7) and 99.4% (98.5, 99.8), respectively. It has excellent diagnostic accuracy.<br>Conclusion: In this review, Sysmex had a better summary estimate of sensitivity and specificity. Therefore, Sysmex is utilized as an alternative diagnostic tool for malaria diagnosis, because, it has excellent diagnostic accuracy.
**Order of Authors:**	Zewudu Mulatie
	Amanuel Kelem
	Bisrat Birke
	Elias Chane
	Amare Mekuanint
	Abebe Yenesew
	Badasa Addisu
	Dereje Mengesha
**Additional Information:**	
**Question**	**Response**

**Financial Disclosure**<br><br>Enter a financial disclosure statement that describes the sources of funding for the work included in this submission. Review the submission guidelines for detailed requirements. View published research articles from *PLOS ONE* for specific examples.<br><br>This statement is required for submission and **will appear in the published article** if the submission is accepted. Please make sure it is accurate.<br><br>**Unfunded studies**<br>Enter: *The author(s) received no specific funding for this work.*<br><br>**Funded studies**<br>Enter a statement with the following details:<br>• Initials of the authors who received each award<br>• Grant numbers awarded to each author<br>• The full name of each funder<br>• URL of each funder website<br>• Did the sponsors or funders play any role in the study design, data collection and analysis, decision to publish, or preparation of the manuscript?<br>• **NO** - Include this sentence at the end of your statement: *The funders had no role in study design, data collection and analysis, decision to publish, or preparation of the manuscript.*<br>• **YES** - Specify the role(s) played.<br><br>* typeset	The author(s) received no specific funding for this work.
**Competing Interests**<br><br>Use the instructions below to enter a competing interest statement for this submission. On behalf of all authors, disclose any competing interests that could be perceived to bias this work—acknowledging all financial support and any other relevant financial or non-financial competing interests.<br><br>This statement is required for submission	The authors have declared that no competing interests exist.

and **will appear in the published article** if the submission is accepted. Please make sure it is accurate and that any funding sources listed in your Funding Information later in the submission form are also declared in your Financial Disclosure statement.<br><br>View published research articles from *PLOS ONE* for specific examples.<br><br>**NO authors have competing interests**<br><br>Enter: *The authors have declared that no competing interests exist.*<br><br>**Authors with competing interests**<br><br>Enter competing interest details beginning with this statement:<br><br>*I have read the journal's policy and the authors of this manuscript have the following competing interests: [insert competing interests here]*<br><br>* typeset	
**Ethics Statement**<br><br>Enter an ethics statement for this submission. This statement is required if the study involved:<br><br>• Human participants<br>• Human specimens or tissue<br>• Vertebrate animals or cephalopods<br>• Vertebrate embryos or tissues<br>• Field research<br><br>Write "N/A" if the submission does not require an ethics statement.<br><br>General guidance is provided below. Consult the submission guidelines for detailed instructions. **Make sure that all information entered here is included in the Methods section of the manuscript.**	N/A

Powered by Editorial Manager® and ProduXion Manager® from Aries Systems Corporation

**Format for specific study types**<br><br>**Human Subject Research (involving human participants and/or tissue)**<br>• Give the name of the institutional review board or ethics committee that approved the study<br>• Include the approval number and/or a statement indicating approval of this research<br>• Indicate the form of consent obtained (written/oral) or the reason that consent was not obtained (e.g. the data were analyzed anonymously)<br><br>**Animal Research (involving vertebrate animals, embryos or tissues)**<br>• Provide the name of the Institutional Animal Care and Use Committee (IACUC) or other relevant ethics board that reviewed the study protocol, and indicate whether they approved this research or granted a formal waiver of ethical approval<br>• Include an approval number if one was obtained<br>• If the study involved *non-human primates*, add *additional details* about animal welfare and steps taken to ameliorate suffering<br>• If anesthesia, euthanasia, or any kind of animal sacrifice is part of the study, include briefly which substances and/or methods were applied<br><br>**Field Research**<br><br>Include the following details if this study involves the collection of plant, animal, or other materials from a natural setting:<br>• Field permit number<br>• Name of the institution or relevant body that granted permission	
**Data Availability**<br><br>Authors are required to make all data underlying the findings described fully available, without restriction, and from the time of publication. PLOS allows rare exceptions to address legal and ethical concerns. See the PLOS Data Policy and FAQ for detailed information.	Yes - all data are fully available without restriction

A Data Availability Statement describing where the data can be found is required at submission. Your answers to this question constitute the Data Availability Statement and **will be published in the article**, if accepted.<br><br>**Important:** Stating 'data available on request from the author' is not sufficient. If your data are only available upon request, select 'No' for the first question and explain your exceptional situation in the text box.<br><br>Do the authors confirm that all data underlying the findings described in their manuscript are fully available without restriction?	
**Describe where the data may be found in full sentences. If you are copying our sample text, replace any instances of XXX with the appropriate details.**<br><br>• If the data are **held or will be held in a public repository**, include URLs, accession numbers or DOIs. If this information will only be available after acceptance, indicate this by ticking the box below. For example: *All XXX files are available from the XXX database (accession number(s) XXX, XXX.).*<br>• If the data are all contained **within the manuscript and/or Supporting Information files**, enter the following: *All relevant data are within the manuscript and its Supporting Information files.*<br>• If neither of these applies but you are able to provide **details of access elsewhere**, with or without limitations, please do so. For example:<br><br>*Data cannot be shared publicly because of [XXX]. Data are available from the XXX Institutional Data Access / Ethics Committee (contact via XXX) for researchers who meet the criteria for access to confidential data.*<br><br>*The data underlying the results presented in the study are available from (include the name of the third party*	All relevant data are within the manuscript and its Supporting Information files.

and contact information or URL).<br>• This text is appropriate if the data are owned by a third party and authors do not have permission to share the data.<br><br>* typeset	
Additional data availability information:	

Manuscript
Click here to access/download;Manuscript;malaria manuscript of sysmex.docx

Diagnostic role of Sysmex hematology analyzer in the detection of malaria: A systematic review and meta-analysis

Zewudu Mulatie[1*], Amanuel Kelem [2], , Elias Chane [3], Amare Mekuanint Tarekegn [3], Bisrat Birke Teketelew[5], Abebe Yenesew [4], Abatneh Melkamu [4], , Yalew Muchie [4], Bedasa Addisu[2], Dereje Mengesha Berta [5]

[1]Department of Medical Laboratory Sciences, College of Medicine and Health Sciences, Wollo University, Dessie, Ethiopia

[2]Department of Medical Laboratory Sciences, Asrat Woldeyes Health Science Campus, Debre Berhan University, Debre Berhan, Ethiopia

[3]Department of Clinical Chemistry, School of Biomedical and Laboratory Science, College of Medicine and Health Sciences, University of Gondar, Gondar, Ethiopia

[4]Department of Medical Laboratory Science, College of Medicine and Health Sciences Debre Markos University, Ethiopia

[5]Department Hematology and immunohematology, School of Biomedical and laboratory science, college of medicine and health science, University of Gondar, Gondar, Ethiopia.

*** Correspondence: Zewudu Mulatie**

Phone number: +251945274251;

Email: zewudumulatie@gmail.com

Authors email

Zewudu Mulatie: zewudumulatie@gmail.com

Amanuel Kelem: amanuelkelem@gmail.com

Bisrat Birke: bisratbirke1921@gmail.com

Elias Chane: eliaschane236@gmail.com

Amare mekuanint :amare.2429.mekuanint@gmail.com

Abebe Yenesew: abebeyenesew2008@gmail.com

Badasa Addisu: bedasaddisu78@gmail.com

Dereje Mengesha: mengeshad826@gmail.com

Abstract

Background: Malaria control depends primarily on rapid and accurate diagnosis followed by successful treatment. Light microscopy is still using as a gold standard method for diagnosis of malaria. Sysmex hematology analyzer is the novel method for malaria detection. Therefore, the aim of this review was to investigate the diagnostic accuracy of Sysmex hematology analyzer for malaria diagnosis.

Methods: Electronic databases like PubMed, PubMed Central, Science Direct databases, Google Scholar, and Scopus were used to find relevant articles. The studies' methodological quality was assessed using the Quality Assessment of Diagnostic Accuracy Studies -2 tool. Using Review Manager 5.4.1, the estimates of sensitivity and specificity, as well as their 95% confidence intervals, were shown in forest plots. Midas software in Stata14.0 was utilized to calculate the summary estimates of sensitivity, specificity, positive likelihood ratio, negative likelihood ratio, and diagnostic odds ratio. Heterogeneity was considerable or the I^2 was greater than 50%, subgroup and meta- regression were performed.

Results: A total of 15 studies were assessed for diagnostic accuracy. The sensitivity and specificity of Sysmex hematology analyzer for studies ranged from 46% to 100% and 81% to 100%, respectively. The summary estimate of sensitivity and specificity of Sysmex hematology analyzer were 95% (95% CI: 85%-99%) and 99% (95% CI: 97%-100%), respectively. It had excellent diagnostic accuracy. There were significant heterogeneity among the studies included in this meta-analysis. The summary estimate of sensitivity and specificity of Sysmex hematology analyzer using polymerase chain reaction as the gold standard was 97.6% (95% CI: 83.2, 99.7) and 99.4% (98.5, 99.8), respectively.

Conclusion: In this review, Sysmex hematology analyzer had better summary estimate of sensitivity and specificity. Therefore, Sysmex hematology analyzer is utilized as an alternative diagnostic tool for malaria diagnosis.

Keywords: Diagnostic test accuracy, malaria, plasmodium species, Sysmex hematology analyzer

Systematic review registration PROSPERO **(**2023: CRD42023427713**).**

Introduction

Malaria continues to pose a significant global public health challenge, with a substantial impact on infection rates and mortality. In 2021, it was estimated that there were approximately 247 million cases of malaria infection and 619,000 reported deaths. The majority of these cases and deaths were concentrated in 29 out of the 84 countries where malaria is endemic, accounting for about 96% of global malaria cases and mortality. Particularly, Africa experienced a large proportion of the burden, with nearly 95% of cases and 96% of deaths occurring in the continent. Among these deaths, approximately 78.9% were children under the age of five. The countries who had the highest number of malaria cases were Nigeria (26.6%), the Democratic Republic of the Congo (12.3%), Uganda (5.1%), and Mozambique (4.1%), collectively contributing to almost half of all cases. Furthermore, four countries - Nigeria (31.3%), the Democratic Republic of the Congo (12.6%), the United Republic of Tanzania (4.1%), and Niger (3.9%) - accounted for slightly over half of all malaria-related deaths worldwide (1). Early and precise diagnosis is a crucial aspect of malaria control and eradication efforts.

The World Health Organization (WHO) emphasizes the importance of confirming the diagnosis of suspected malaria cases through either microscopy or a rapid diagnostic test (RDT) before initiating treatment (2). Malaria diagnosis is typically conducted by visually identifying the parasite under a microscope, which is considered the most reliable diagnostic method in clinical settings. This approach is cost-effective, allows for the quantification of parasitemia, facilitates follow-up during parasitemia clearance, and offers the potential for detecting other diseases. However, microscopic diagnosis is time-consuming, requires skilled personnel, and has limitations associated with the expertise of the observer and its low limit of detection (3).

Conversely, rapid diagnostic tests (RDTs) require less training, offer convenience, and provide results within a short timeframe of approximately fifteen minutes. As a result, RDTs have effectively replaced microscopy in routine diagnosis within health institutions, as well as for active case identification by community health workers, particularly in rural areas. However, RDTs do have limitations in that they are not quantitative and cannot distinguish between different stages of parasite development. Consequently, RDTs have limited utility in assessing treatment efficacy, monitoring progression, and assessing their impact on transmission (4). In addition, recent reports stated that the RDT cause false negatives in Plasmodium falciparum due

to deletions of the gene encoding target histidine rich protein 2/3 (HRP2/3) (5-7). Plasmodium lactate dehydrogenase (pLDH) detecting RDTs are also available, however they have lesser sensitivity than HRP2-detecting RDTs (8).

Nucleic acid amplification techniques (NAATs), including polymerase chain reaction (PCR), offer superior sensitivity compared to other methods, with reported detection limits as low as 5 parasites per microliter (9-11). However, these techniques are costly and time-consuming, requiring trained personnel for operation. Additionally, they do not provide useful information for follow-up after treatment and cannot differentiate between different stages of the parasite (3).

In malaria-endemic regions, there is a demand for improved diagnostic methods that can complement existing approaches, particularly those that offer high sensitivity and ease of use. The automated Sysmex hematology analyzer is an innovative and clinical tool for malaria diagnosis. This advanced technology reduces analysis time and improves accuracy in detecting potential malaria infections. It offers fast, sensitive, and cost-effective examinations for efficient malaria screening (12-14). The analyzer works by combining optical fluorescence methods, flow cytometry, and laser-optical recognition. The Sysmex hematology analyzer provides a complete blood count (CBC) and classifies distinct blood cell types, as well as directly detects and quantifies plasmodium parasites in blood in one minute and had high a limit of quantification (13).

Affordability of equipment, uantity of tests to be conducted, skilled staff, and diagnostic procedure accuracy are the factors affect the selection of diagnostic tests. However, even if a test method is practical and economical, and sometimes the only option in a given case, it must be accurate enough (15). Hence, the objective of this review was to assess the diagnostic accuracy of the Sysmex hematology analyzer for malaria diagnosis.

Methods

Search strategy and Study selection

This systematic review and meta-analysis followed the PRISMA guidelines for reporting. Several electronic databases, including PubMed, PubMed Central, Science Direct, Google Scholar, and Scopus, were searched for relevant studies from April through June 14, 2023. The search utilized keywords such as "malaria," "P. falciparum," "P. vivax," "P. ovale," "P.

malariae," "diagnosis," "Sysmex hematology analyzer," and "diagnostic test accuracy," with Boolean operators (AND, OR) used to combine the keywords. Overlapping studies across multiple databases were excluded, and additional papers were found through the reference lists of the included studies. Duplicate studies and studies without a reference test were excluded, and the title and abstract of potentially acceptable research were independently screened by four reviewers. The full text of potentially eligible studies reporting the diagnostic accuracy of the Sysmex hematology analyzer was then assessed for extraction, with any disagreements among authors resolved through discussion.

Eligibility criteria

This review included original publications in English that assessed the diagnostic accuracy of the Sysmex hematology analyzer for detecting plasmodium species. Studies involving non-human subjects (animals, rodents), review papers, case studies, and letters to the editor were excluded. This review included original publications that evaluated the diagnostic accuracy of the Sysmex hematology analyzer haematology analyzer for the detection of plasmodium species. Only studies reported in English were considered. Studies reporting the diagnostic accuracy of the Sysmex hematology analyzer for the detection of malaria in non-human subjects (animals, rodents) were excluded. Review papers, case studies, and letters to the editor were also excluded.

Data extraction and management

Data from eligible papers were independently extracted by three reviewers in Microsoft Excel 2010, including information on authors, publication year, region, study subjects, study design, descriptions of reference and index tests, and data for 2x2 tables. The methodological quality of the included studies was assessed using the QUADAS-2 tool, evaluating bias in patient selection, index test, reference standards, and flow/timing (16). A graph illustrating the risk of bias was created.

Statistical analysis

Statistical analyses were conducted using Review Manager 5.4 for the estimates of sensitivity and specificity, as well as their 95% confidence intervals were shown in forest plots. Midas in Stata14 used for to assess the summary estimates of sensitivity, specificity, positive likelihood ratio, negative likelihood ratio, and diagnostic odds ratio (DOR) were calculated. The hierarchical summary receiver operating characteristic curve (HSROC) was used to assess heterogeneity. Meta-disc 1.4.0 software was employed to determine the presence of a threshold

effect if heterogeneity was significant or I^2 exceeded 50%. Meta-regression was explored to identify sources of heterogeneity, such as sampling method, study population, sample size, reference method, type of Sysmex hematology analyzer, and parameter detected. Deeks' funnel plot asymmetry test was performed to assess publication bias.

Results

Description of included studies

Database searches yielded a total of 936 articles, of which 612 were removed due to duplication. A total of 324 papers were screened for their titles and abstracts, and 304 studies were excluded. Among 20 full-text articles reviewed against the eligibility criteria, then 5 full-text articles were excluded. Finally, fifteen articles were eligible and included in the systematic review and meta-analysis of the final analysis (Figure 1).

Figure 1: Flow diagram of the included studies for the systematic review and meta-analysis

Characteristics of the included studies

The systematic review examined fifteen papers published up to 2023, resulting in 16,501 people being evaluated to evaluate the performance of the Sysmex hematology analyzer (17-30). In 9 studies (11,220 tests), polymerase chain reaction was employed as a reference method for Sysmex hematology analyzer (17-24). A microscope was also used as a reference method for 6 Sysmex hematology analyzer testing (5281 tests) (25-30).

Table 1 Characteristics of the studies included in the systematic review and meta-analysis of the performance of Sysmex hematology analyzer

s. no.	Author (year)	Population	Sample size	TP	FP	TN	FN	Sysmex hematology analyzer type	Parameters	Reference test
1	Baya et al (2021) (17)	Malaria suspected	5031	513	41	4477	0	XN-31	MI- RBC	PCR
2	Buoro et al (2018) (25)	Malaria suspected	1061	13	200	847	1	XN series	Diff WBC	microscope
3	Hee et al (2008) (26)	Patients	487	100	0	343	44	XE-2100	Diff WBC	microscope
4	Kagaya et al (2022) (18)	Malaria suspected	169	18	11	135	5	XN-31	MI- RBC	PCR
5	Khartabil et al (2022) (27)	Malaria suspected	112	13	1	96	2	XN-31	MI- RBC	microscope
6	Picot et al (2022) (19)	Febrile patients	357	109	4	244	0	XN-31	MI- RBC	PCR
7	Pillay et al 7[a] (2019) (20)	Malaria suspected	191	124	0	67	0	XN-30	MI- RBC	PCR
8	Pillay et al 7[b](2019) (20)	Malaria suspected	1028	272	1	749	6	XN- 10	MI- RBC	PCR
9	Post et al (2019) (21)	Febrile patients	837	253	2	478	104	XN-30	MI-RBC	PCR
10	Sarita et al (2011) (28)	Malaria suspected	430	52	35	325	18	XE-2100	Diff WBC	microscope
11	Sharma et al (2013) (29)	Malaria suspected	2251	147	129	1974	1	XE-2000i	Diff WBC	microscope
12	Sunilkumar et al (2016) (30)	Febrile patients	940	40	24	867	9	XN 1000	MI- RBC	microscope
13	Yasuda et al (2022) (22)	Malaria suspected	80	32	0	47	1	XN-31	MI- RBC	PCR
14	Yoo et al (2010) (23)	Patients	1801	191	4	1384	222	XE-2100	Diff WBC	PCR
15	Zuluaga et al (2021) (24)	Febrile patients	1726	513	2	1154	57	XN-31	MI- RBC	PCR

Note: NA, not applicable; PCR: polymerase chain reaction; TP, true positive; FP, false positive; FN, false negative; TN: true negative; MI-RBC: malaria infected red blood cell; Diff-WBC: Differential white blood cell

NB; [a or b] : indicates in one study compare 2 types of symex hematology analyzer

Data quality assessment

The risk of bias in patient selection was rated as low in six of the fifteen diagnostic studies and high in two of the fifteen studies. In the applicability concern domain, there was no high risk of bias in patient selection. In the nine studies are the risk of bias in flow and timing was considered low (Figure **2**).

Figure 2: Risk of bias graph of studies included in the meta-analysis

Diagnostic accuracy of Sysmex hematology analyzer

The systematic review and meta-analysis comprised fifteen studies to evaluate the diagnostic accuracy of Sysmex hematology analyzer. For those studies, the sensitivity and specificity ranged from 46% to 100% and 81% to 100%, respectively. For those investigations, the summary estimates of sensitivity and specificity of Sysmex hematology analyzer were 95% (95%, CI: 85% -99%) and 99% (95%, CI: 97% -100%), respectively. Sysmex hematology analyzer had a DOR of 1890 (95% CI: 392-9112), a positive likelihood ratio (LR+) of 94.1 (95% CI: 34.6-256.1), and a negative likelihood ratio (LR-) of 0.05 (95% CI: 0.03-0.07). The area under curve (AUC) was 1.00 (95% CI: 0.99-1.00), indicating that the test had excellent diagnostic accuracy (Figure **3**).

Figure 3 Forest plot of Sysmex hematology analyzer for malaria detection

Sub group analysis

Studies conducted to assess Sysmex hematology analyzer accuracy revealed significant heterogeneity (Q = 416.938, I2 = 100, and P< 0.01). Sub-group analysis was performed based on continent, publication year, study design, study population, reference tests, Sysmex hematology analyzer types, and malaria detection parameters. Pooled sensitivity in febrile patients was 92% (95% CI: 65%, 99%), while pooled sensitivity in malaria patients was 97% (95% CI: 82%, 99%). When PCR was employed as a reference test, Sysmex hematology analyzer had sensitivity and specificity of 98% (95% CI: 83, 100) and 99% (99, 100), respectively. When the microscope test was used as the gold standard, the sensitivity and specificity of Sysmex hematology analyzer were 82% (95% CI: 44%, 96%) and 97% (95% CI: 89%, 99%), respectively. The sensitivity of the Sysmex hematology analyzer XN series was 98% (90%, 99%), while the sensitivity of the Sysmex hematology analyzer XE series was 83% (95%CI: 44%, 97%). The threshold effect and

meta-regression were used to investigate the cause of heterogeneity. There was no threshold effect between Asian studies (P=0.19), between 2016 and 2020 (P=0.28), febrile patients (P=0.07), Sysmex hematology analyzer with microscope (P=0.32), Sysmex hematology analyzer XE series (P=0.33), mi-RBC (P=0.21), and diff-WBC (P=0.38) (Table **2**).

Table 2 Subgroup analysis of Sysmex hematology analyzer

Sub group		No. of studies	Pooled sensitivity (95%CI)	Pooled specificity (95%CI)	Heterogeneity test (I^2)	P-value
Continent	Africa	5	99% (75%, 100%)	99% (0.97%, 100%)	97%	< 0.01*
	Asia	6	86% (62%, 96%)	99% (94%, 100%	99%	0.19
	Total pooled	11	94% (79%,98)	99 (97%, 100%)	99%	<0.01*
Publication year	≤ 2015	4	83% (44%, 97%)	99% (91%, 100%)	99%	0.33
	2016-2020	5	95% (75%, 99%)	99% (92%, 100%)	95%	0.28
	2021-2023	6	97% (55%, 100%)	99% (97%, 100%)	98%	< 0.01*
	Total pooled	15	95% (85%, 99%	99% (97%, 100%)	100%	< 0.01*
Study design	Cross sectional	10	87% (63%, 96%)	99% (97%, 100%)	98%	0.04*
	Prospective	4	99% (45%, 100%)	98% (95%, 100%)	98%	< 0.01*
	Total pooled	14	94% (77%, 99%)	99% (98, 100%)	99%	< 0.01*
Population	Febrile patient	4	92% (65%, 99%)	99% (98%, 100%)	91%	0.07
	Malaria suspected	9	97% (82%, 99%)	98% (95%, 99%)	93%	0.04*
	Total	13	96%(85%, 99)	98% (97%, 99%)	95%	0.03*

	pooled					
Reference tests	PCR	9	98% (83, 100)	99 (99, 100)	99%	< 0.01*
	Microscope	6	82% (44%, 96%)	97% (89%, 99%	99%	0.32
	Total pooled	15	95% (85%, 99%	99% (97%, 100%)	100%	< 0.01*
Sysmex hematology analyzer types	XN series	11	98% (90%, 99%)	99% (98%, 100%)	98%	0.02*
	XE series	4	83% (44%, 97%)	99% (91%, 100)	99%	0.33
	Total pooled	15	95% (85%, 99%	99% (97%, 100%)	100%	< 0.01*
Parameters	mi-RBC	10	96% (77%, 99%)	99% (98%, 99%)	97%	0.21
	Diff WBC	5	86% (56%, 97%)	98% (86%, 100%)	99%	0.38
	Total pooled	15	95% (85%, 99%	99% (97%, 100%)	100%	< 0.01*

Note; *statistically signifcant

The meta-regression of those studies revealed that the most significant sources of heterogeneity (P<0.05) were the reference test, Sysmex hematology analyzer types, and malaria detection parameters (Table 3).

Table 3 Meta-regression analysis of diagnostic accuracy

Variables	Coefficient	Standard error	p-value	RDOR	95%CI
Ste	6.12	1.41	<0.01	NA	NA
S	0.43	0.12	0.03	NA	NA
Study population	-0.08	0.76	0.89	0.65	0.12–5.31
Sample size	−1.45	0.57	0.03*	0.49	0.02–0.94
Reference test	1.09	0.3541	0.04*	2.15	1.19–19.03
Sysmex	1.67	0.5436	0.02*	3.46	1.32–15.91

hematology analyzer types					
Parameters	1.81	0.6310	0.01*	4.29	1.61–19.67

*statistically signifcant NA, not applicable; CI, confdence interval; RDOR, relative diagnostic odds ratio; Ste, constant term in the equation; S, a measure of threshold

Sysmex hematology analyzer with PCR and microscope as reference test

The sensitivity and specificity of Sysmex hematology analyzer performed with PCR as a reference test were varies from 46%-100% and 92%-100%, respectively. Six of the studies (66.7%, 6/9) demonstrated a sensitivity of 90% and more. Eight (88.9%, 8/9) studies showed a specificity ≥95%. The sensitivity and specificity of Sysmex hematology analyzer done with microscope as reference test ranges from 69%–99% to 81%–100%, respectively. Two (2/6) studies showed a sensitivity ≥90%. Three (50%, 3/6) studies showed a specificity ≥95% (Figure **4**).

Figure 4 Forest plot of Sysmex hematology analyzer with PCR and microscope as reference test

The summary estimate of sensitivity and specificity of Sysmex hematology analyzer were 97.6% (95% CI: 83.2, 99.7) and 99.4% (95% CI: 98.5, 99.8), respectively, using PCR as reference test. It had a DOR of 7880.01 (95% CI: 791.03, 78498.74), a positive likelihood ratio (LR+) of 186.87 (95% CI: 67.93, 514.07), and a negative likelihood ratio (LR-) of 0.023 (95% CI: 0.003, 0.188). The area under the curve was 1.00 (95% CI: 0.99-1.00), indicating that the test had excellent diagnostic accuracy (Figure **5**).

Figure 5: Summary receiver operating characteristic plot of sensitivity and specificity of Sysmex hematology analyzer with PCR as a reference test

Sysmex hematology analyzer with microscope as reference

The summary estimate of sensitivity and specificity of employing a microscope as the gold standard method were estimated to be 81.7% (95% CI: 44%, 96) and 97.02% (95% CI: 88.92, 99.7), respectively. With a microscope as the gold standard, the diagnostic odds ratio, LR+, and LR- of symex were 146.27 (95% CI: 29.86, 716.4), 27.50 (95% CI: 8.03, 94.20), and 0.188 (95% CI: 0.046, 0.763), respectively. The AUC was 0.98 (95% CI: 0.96-0.99), indicating that the test had excellent diagnostic accuracy (Figure **6**).

Figure 6: Summary receiver operating characteristic plot of sensitivity and specificity of Sysmex hematology analyzer with microscope as a reference test

Publication bias

The Deeks' funnel plot asymmetry test of DOR revealed no significant asymmetry (P = 0.14), indicating that there was no observable publication bias (Figure **7**).

Figure 7 Publication bias of Sysmex hematology analyzer for diagnostic accuracy

Discussion

An accurate and timely diagnosis is critical for managing, controlling, and eradicating malaria. Early malaria diagnosis is necessary to guide adequate treatment and decrease the severe effects of infection (1). Lack of clinical and laboratory experience, prolonged incubation periods and plasmodium vivax relapses (31, 32), or prophylaxis in travellers can all delay diagnosis, increasing the risk of malaria-related complication (33). An accurate malaria detection technology integrated into routine Sysmex hematology analyzer could assist detect infections earlier and potentially reduce negative consequences associated with malaria infection. It also utilized by minimally trained personnel, not requires sample preparation, has no observer-dependent viability, and is available worldwide. Another significant advantage of the Sysmex hematology analyzer automation is that it delivers a CBC with each assay, integrating malaria diagnosis and treatment (12).

All accessible studies, both symptomatic and asymptomatic, were extensively searched. The majority of these investigations were undertaken after 2015; only one study was conducted before 2010, reflecting the relatively recent interest in this diagnostic approach. The I^2 statistic was used for investigating statistical heterogeneity, which reflects variance across studies due to inter-study variability. Heterogeneity was expected to be associated to the test reading method, the patients, parasitaemia levels, and the comparators. There was significant heterogeneity among the studies included in this meta-analysis. Meta-regression analysis for Sysmex hematology analyzer revealed that the gold standard reference test, Sysmex hematology analyzer types and parameters for malaria detection were the sources of heterogeneity.

The overall estimate sensitivity and specificity of Sysmex hematology analyzer were 95% (95%CI: 85-99%) and 99% (95%CI: 97%-100%), respectively, according to this review. The Sysmex hematology analyzer had high diagnostic accuracy (AUC: 1.00 (95% CI: 0.99–1.00). Sysmex hematology analyzer had 1890 (95% CI 392-9112) times higher odds of obtaining positive result in diseased persons than in non-diseased one. On the other hand, it showed 94.1 (95% CI: 34.6–256.1) times higher odds of positive test in positive individuals than negative individuals. As a result, Sysmex hematology analyzer is utilized as an alternative diagnostic tool for malaria diagnosis. Because of has high diagnostic accuracy.

This review's pooled sensitivity was higher than the sensitivity of studies on RDT diagnostic accuracy (42% for conventional vs. 61% for ultrasensitive RDT) (34), while another study revealed lower sensitivity (44.3% for conventional and 56.1% for high sensitive RDT) (35). The reduced sensitivity of RDT may be attributed to asymptomatic malaria and deletions of the Plasmodium falciparum gene encoding the target histidine rich protein 2/3 (HRP2/3). Furthermore, the microscope's diagnostic accuracy in pregnancy-associated malaria exhibited low sensitivity (70.8%) (36). It could be because microscopes require expert laboratory personnel, have limited diagnostic accuracy, and generate false negative results in asymptomatic malaria. This review's pooled sensitivity was consistent with a systematic review and meta-analysis of the diagnostic accuracy of loop-mediated isothermal amplification (LAMP) techniques, which indicated a sensitivity of 97% (37) and another review, which stated a sensitivity of PCR was 98% (38). The pooled specificity of Sysmex hematology analyzer had high specificity in this review and was almost identical to the specificity of RDT (34), microscope (36), LAMP (37) and PCR (38).

In this review, Sysmex hematology analyzer with PCR as gold standard shown higher pooled sensitivity than Sysmex hematology analyzer with microscopy as gold standard (97.6% (95% CI: 83.2-99.7%) vs. 81.7% (95% CI: 44%, 96). However, the overall estimate of specificity of Sysmex hematology analyzer using PCR as the reference method and Sysmex hematology analyzer utilising microscopy as the gold standard method had comparable specificity (99.4% (95% CI: 98.5, 99.8) vs. 97.02% (95% CI: 88.92%, 99.7%), respectively. Also, the diagnostic accuracy of Sysmex hematology analyzer with PCR as gold standard and Sysmex hematology

analyzer with microscope was almost similar (AUC: 1.00 (95% CI: 0.99-1.00) vs. 0.98 (95% CI: 0.96-0.99)), respectively.

The sensitivity of Sysmex hematology analyzer with PCR as gold standard was greater in this review than a systematic review and meta-analysis study on microscope with PCR in Ethiopia, which showed a sensitivity of 75.20% (15), and another review in Colombia, which stated a sensitivity of 70.8% (36). In the other hand, the specificity of this study was nearly identical to that of a studies on microscopy with PCR in Ethiopia (97.12%) (15) and Colombia (99%) (36). The summary estimates of sensitivity this review higher than the review done about RDT with PCR in Ethiopia were 66.18% (15). In contrast, the summary estimations of specificity of this review were similar to review done in Ethiopia (95.36%) (15). This review's sensitivity was almost identical to that of a study on LAMP using PCR (97%)(37).

The sensitivity of Sysmex hematology analyzer with microscope as reference test was lower in this review than in a systematic review and meta-analysis study on RDT with microscope in Ethiopia (95.05%) (15) and India (97.0%) (39). The lower sensitivity for this review could be attributed to the fact that the majority of the studies used differential WBC scatter for malaria diagnosis. According to a review of haematology analyzers for malaria detection, the decrease in sensitivity for differential WBC abnormalities could be due to the lack of a consensus definition for this diagnosis criterion, as well as difficulty and subjectivity in manually evaluating these patterns, which could have resulted in a classification bias. However, the specificity of this study was nearly same to that of review conducted in Ethiopia (96.47%) (15) and India (96.0%) (39).

The sensitivity of Sysmex hematology analyzer XN series was higher compared to XE series (98% vs. 83%), respectively in this review. The Sysmex hematology analyzer XN series and XE series types had similar specificity (99%). Both Sysmex hematology analyzer XN series and XE series types had excellent diagnostic accuracy (AUC: 0.99 (95%CI: 0.99-1.00) vs. 0.99 (95%CI: 0.97-0.99)).

In this review, the sensitivity of Sysmex hematology analyzer by using malaria infected RBC for malaria detection was higher than the use of diff WBC (96% vs. 86%), respectively. The specificity of Sysmex hematology analyzer using mi-RBC and diff-WBC had almost similar (99% vs.98%), respectively. Generally, both Sysmex hematology analyzer with mi- RBC and

diff- WBC techniques had excellent diagnostic accuracy (AUC: 0.99 (95%CI: 0.98-1.00) vs. 0.98 (95%CI: 0.96-0.99)).

Conclusion

Sysmex hematology analyzer had high summary estimate of sensitivity and specificity. The Pooled sensitivity was higher for Sysmex hematology analyzer using PCR as the gold standard than for Sysmex hematology analyzer using a microscope as the gold standard. As a result, Sysmex hematology analyzer is used as an alternate diagnostic tool for malaria diagnosis due to its high diagnostic accuracy. It also has a low cost, the ability to quantify parasite density and monitor treatment response, can be used by minimally trained staff, does not require sample preparation, has no observer-dependent variability, and provides a CBC with each analysis, synchronising malaria diagnosis and management.

Abbreviation

- AUC: Area Under Curve
- CBC: Complete Blood cell Count
- DOR: Diagnostic Odd Ratio
- LAMP : Loop-mediated isothermal Amplification
- MI-RBC: Malaria Infected Red Blood Cell
- PCR: Polymerase-Chain-Reaction
- RDT: Rapid Diagnostic Test
- WBC: White Blood Cell
- WHO: World health organization

Acknowledgement

The authors would like to express their gratitude to the authors of the included research as well as the participants in their investigations.

Author contribution

The study was conceptualised, developed and manuscript drafted by ZM. Article search and data extraction were carried out by ZM, DMB, EC, AK, AMT and BBT. Quality assessment of the

included studies conducted by ZM, AY and AM. Statistical analysis and interpretation of the data were performed by ZM, EA, DMB and YM. To check the validity and monitor the overall process critically reviewed by ZM, DMB and BA. All the authors read and approved the final manuscript.

Availability of data and materials

The article contains all of the data needed for this study. If more information is required, it can be obtained from the associated authors upon request.

Funding

There is no specific funding for this review**.**

Declaration

Ethical approval and consent to participate

Ethical approval and consent to participate are not required because this is a systematic review and meta-analysis that does not involve human or animal experiments.

Consent for publication

Not applicable

Competing interests

The authors declared that there is no competing interest.

Author details

Authors' information

Z.M and EA has a Masters in Hematology & Immunohematology unit from Department of Medical Laboratory Sciences of Wollo University Dessie, Ethiopia. DMB and BBT have a Masters in Hematology & Immunohematology department in School of Biomedical and Laboratory science, College of Medicine and Health sciences, University of Gondar. EC and AMT have a Masters in department of Clinical Chemistry, School of Biomedical and Laboratory Science, College of Medicine and Health Sciences, University of Gondar, Gondar, Ethiopia. AY,AM and YM have a masters in department of Medical Laboratory Science, College of Medicine and Health Sciences Debre Markos University, Ethiopia; AK and BA have masters in department of Medical Laboratory Sciences, Asrat Woldeyes Health Science Campus, Debre Berhan University, Debre Berhan, Ethiopia

Reference

1. World Health Organization. World Malaria Report 2022. 2022.

2. World Health Organization. Global Technical Strategy for Malaria 2016–2030. 2015.

3. Mathison BA, Pritt BS. Update on Malaria Diagnostics and Test Utilization. J Clin Microbiol. 2017;55(7):2009-17.

4. Boyce MR, O'Meara WP. Use of malaria RDTs in various health contexts across sub-Saharan Africa: a systematic review. BMC Public Health. 2017;17(1):470.

5. Verma AK, Bharti PK, Das A. HRP-2 deletion: a hole in the ship of malaria elimination. Lancet Infect Dis. 2018;18(8):826-7.

6. Watson OJ, Slater HC, Verity R, Parr JB, Mwandagalirwa MK, Tshefu A, et al. Modelling the drivers of the spread of Plasmodium falciparum hrp2 gene deletions in sub-Saharan Africa. Elife. 2017;6.

7. Beshir KB, Sepúlveda N, Bharmal J, Robinson A, Mwanguzi J, Busula AO, et al. Plasmodium falciparum parasites with histidine-rich protein 2 (pfhrp2) and pfhrp3 gene deletions in two endemic regions of Kenya. Sci Rep. 2017;7(1):14718.

8. Abba K, Kirkham AJ, Olliaro PL, Deeks JJ, Donegan S, Garner P, et al. Rapid diagnostic tests for diagnosing uncomplicated non-falciparum or Plasmodium vivax malaria in endemic countries. Cochrane Database Syst Rev. 2014;2014(12):Cd011431.

9. Zimmerman PA, Howes RE. Malaria diagnosis for malaria elimination. Curr Opin Infect Dis. 2015;28(5):446-54.

10. Hofmann NE, Gruenberg M, Nate E, Ura A, Rodriguez-Rodriguez D, Salib M, et al. Assessment of ultra-sensitive malaria diagnosis versus standard molecular diagnostics for malaria elimination: an in-depth molecular community cross-sectional study. Lancet Infect Dis. 2018;18(10):1108-16.

11. Wang B, Han SS, Cho C, Han JH, Cheng Y, Lee SK, et al. Comparison of microscopy, nested-PCR, and Real-Time-PCR assays using high-throughput screening of pooled samples for diagnosis of malaria in asymptomatic carriers from areas of endemicity in Myanmar. J Clin Microbiol. 2014;52(6):1838-45.

12. Campuzano-Zuluaga G, Hänscheid T, Grobusch MP. Automated haematology analysis to diagnose malaria. Malar J. 2010;9:346.
13. Tougan T, Suzuki Y, Itagaki S, Izuka M, Toya Y, Uchihashi K, et al. An automated haematology analyzer XN-30 distinguishes developmental stages of falciparum malaria parasite cultured in vitro. Malar J. 2018;17(1):59.
14. Tougan T, Suzuki Y, Izuka M, Aono K, Okazaki T, Toya Y, et al. Application of the automated haematology analyzer XN-30 in an experimental rodent model of malaria. Malar J. 2018;17(1):165.
15. Feleke DG, Alemu Y, Yemanebirhane N. Performance of rapid diagnostic tests, microscopy, loop-mediated isothermal amplification (LAMP) and PCR for malaria diagnosis in Ethiopia: a systematic review and meta-analysis. Malar J. 2021;20(1):384.
16. Whiting P, Rutjes AW, Reitsma JB, Bossuyt PM, Kleijnen J. The development of QUADAS: a tool for the quality assessment of studies of diagnostic accuracy included in systematic reviews. BMC Med Res Methodol. 2003;3:25.
17. M'Baya B, Mfune T, Samon A, Hwandih T, Münster M. Evaluation of the Sysmex XN-31 automated analyser for blood donor malaria screening at Malawi Blood Transfusion Services. Vox Sang. 2022;117(3):346-53.
18. Kagaya W, Takehara I, Kurihara K, Maina M, Chan CW, Okomo G, et al. Potential application of the haematology analyser XN-31 prototype for field malaria surveillance in Kenya. Malar J. 2022;21(1):252.
19. Picot S, Perpoint T, Chidiac C, Sigal A, Javouhey E, Gillet Y, et al. Diagnostic accuracy of fluorescence flow-cytometry technology using Sysmex XN-31 for imported malaria in a non-endemic setting. Parasite. 2022;29:31.
20. Pillay E, Khodaiji S, Bezuidenhout BC, Litshie M, Coetzer TL. Evaluation of automated malaria diagnosis using the Sysmex XN-30 analyser in a clinical setting. Malar J. 2019;18(1):15.
21. Post A, Kaboré B, Reuling IJ, Bognini J, van der Heijden W, Diallo S, et al. The XN-30 hematology analyzer for rapid sensitive detection of malaria: a diagnostic accuracy study. BMC Med. 2019;17(1):103.
22. Komaki-Yasuda K, Kutsuna S, Kawaguchi M, Kamei M, Uchihashi K, Nakamura K, et al. Clinical performance testing of the automated haematology analyzer XN-31 prototype using whole blood samples from patients with imported malaria in Japan. Malar J. 2022;21(1):229.

23. Yoo JH, Song J, Lee KA, Sun YK, Kim YA, Park TS, et al. Automated detection of malaria-associated pseudoeosinophilia and abnormal WBC scattergram by the Sysmex XE-2100 hematology analyzer: a clinical study with 1,801 patients and real-time quantitative PCR analysis in vivax malaria-endemic area. Am J Trop Med Hyg. 2010;82(3):412-4.
24. Zuluaga-Idárraga L, Rios A, Sierra-Cifuentes V, Garzón E, Tobón-Castaño A, Takehara I, et al. Performance of the hematology analyzer XN-31 prototype in the detection of Plasmodium infections in an endemic region of Colombia. Sci Rep. 2021;11(1):5268.
25. Buoro S, Manenti B, Seghezzi M, Moioli V, Bagorria M, Callegaro A, et al. Abnormal scattergrams and cell population data generated by fully automated hematological analyzers: New tools for screening malaria infection? Int J Lab Hematol. 2018;40(3):326-34.
26. Huh HJ, Oh GY, Huh JW, Chae SL. Malaria detection with the Sysmex XE-2100 hematology analyzer using pseudoeosinophilia and abnormal WBC scattergram. Ann Hematol. 2008;87(9):755-9.
27. Khartabil TA, de Rijke YB, Koelewijn R, van Hellemond JJ, Russcher H. Fast detection and quantification of Plasmodium species infected erythrocytes in a non-endemic region by using the Sysmex XN-31 analyzer. Malar J. 2022;21(1):119.
28. Mohapatra S, Samantaray JC, Arulselvi S, Panda J, Munot K, Saxena R. Automated detection of malaria with haematology analyzer Sysmex XE-2100. Indian J Med Sci. 2011;65(1):26-31.
29. Sharma S, Sethi N, Pujani M, Kushwaha S, Sehgal S. Abnormal WBC scattergram: a clue to the diagnosis of malaria. Hematology. 2013;18(2):101-5.
30. Sunilkumar K, Naik P. Usefulness of automated hematology analyzer Sysmex XN 1000 in detection of Malaria. Indian J Path Onc. 2016;3:658-61.
31. Pinter E, Szabo O, Tdorova R, Varnai Z, Banhegyi D. Relapse of Plasmodium vivax and recrudescence of Plasmodium malariae malaria as detectes by Sysmex XE 2100 fully automated blood cell analyzer. Sysmex Journal International. 2007;17(2).
32. Durante Mangoni E, Severini C, Menegon M, Romi R, Ruggiero G, Majori G. Case report: An unusual late relapse of Plasmodium vivax malaria. Am J Trop Med Hyg. 2003;68(2):159-60.
33. Newman RD, Parise ME, Barber AM, Steketee RW. Malaria-related deaths among U.S. travelers, 1963-2001. Ann Intern Med. 2004;141(7):547-55.

34. Yimam Y, Mohebali M, Abbaszadeh Afshar MJ. Comparison of diagnostic performance between conventional and ultrasensitive rapid diagnostic tests for diagnosis of malaria: A systematic review and meta-analysis. PLoS One. 2022;17(2):e0263770.
35. Ochola LB, Vounatsou P, Smith T, Mabaso ML, Newton CR. The reliability of diagnostic techniques in the diagnosis and management of malaria in the absence of a gold standard. Lancet Infect Dis. 2006;6(9):582-8.
36. Gómez-Hoyos R, Cardona-Arias JA, Higuita Gutiérrez LF, Salas-Zapata W, Carmona-Fonseca J. Systematic review of the diagnostic accuracy of thick smear compared to polymerase chain reaction for pregnancy-associated malaria, 2010-2022. Rev Peru Med Exp Salud Publica. 2022;39(3):302-11.
37. Picot S, Cucherat M, Bienvenu AL. Systematic review and meta-analysis of diagnostic accuracy of loop-mediated isothermal amplification (LAMP) methods compared with microscopy, polymerase chain reaction and rapid diagnostic tests for malaria diagnosis. Int J Infect Dis. 2020;98:408-19.
38. Roth JM, Korevaar DA, Leeflang MM, Mens PF. Molecular malaria diagnostics: A systematic review and meta-analysis. Crit Rev Clin Lab Sci. 2016;53(2):87-105.
39. Kojom Foko LP, Pande V, Singh V. Field Performances of Rapid Diagnostic Tests Detecting Human Plasmodium Species: A Systematic Review and Meta-Analysis in India, 1990-2020. Diagnostics (Basel). 2021;11(4).

Click here to access/download;Figure;Fig.1..tif

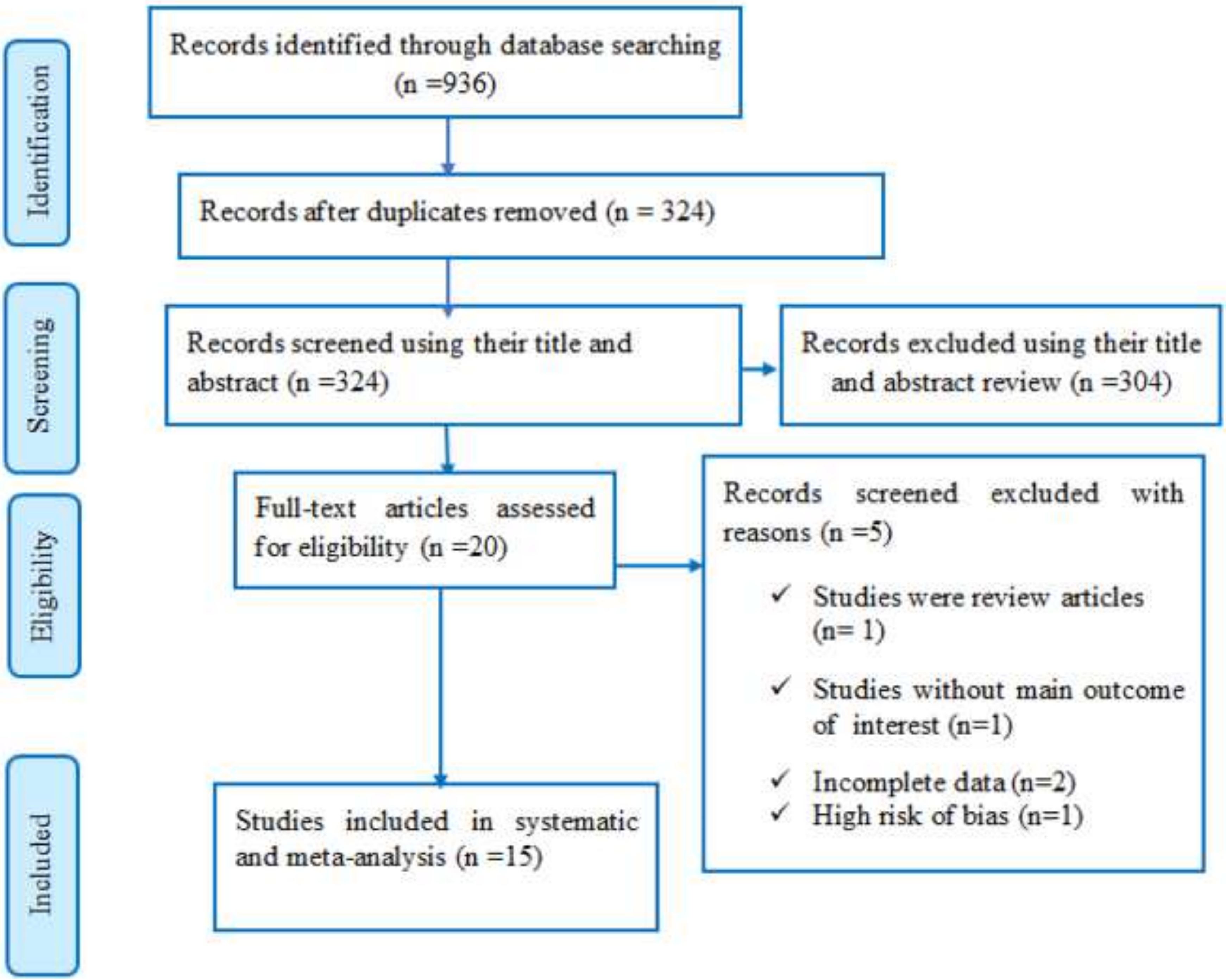

Click here to access/download;Figure;Fig.2..tif

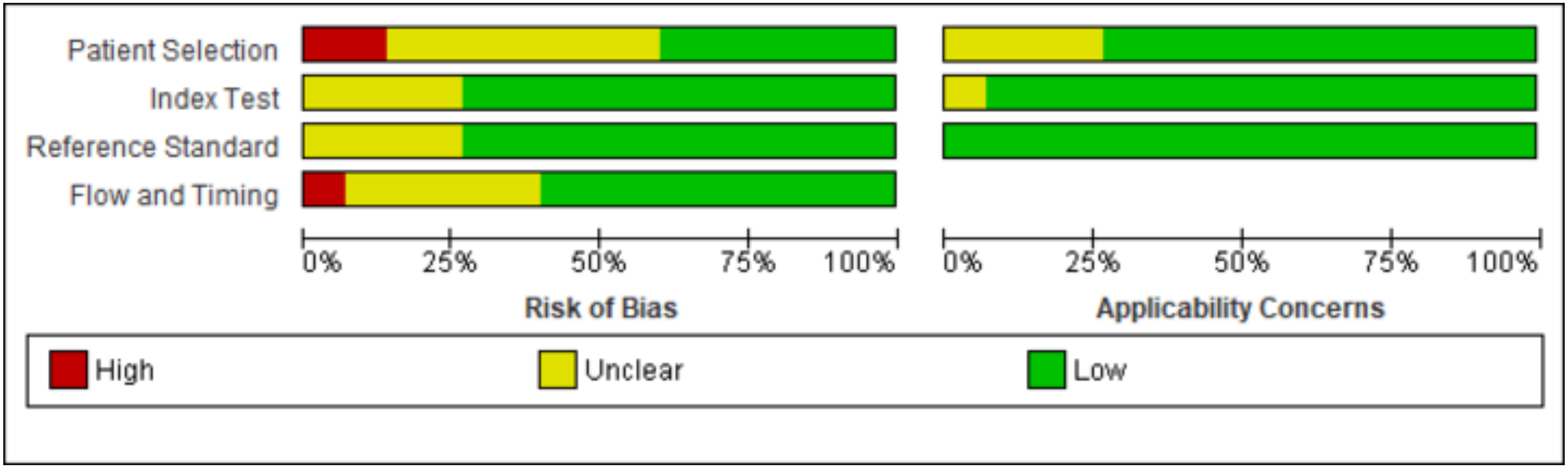

Click here to access/download;Figure;Fig.3..tif

Study	TP	FP	FN	TN	Sensitivity (95% CI)	Specificity (95% CI)
Baya et al 2021	513	41	0	4477	1.00 [0.99, 1.00]	0.99 [0.99, 0.99]
Buoro et al 2018	13	200	1	847	0.93 [0.66, 1.00]	0.81 [0.78, 0.83]
Hee et al 2008	100	0	44	343	0.69 [0.61, 0.77]	1.00 [0.99, 1.00]
Kagaya et al 2022	18	11	5	135	0.78 [0.56, 0.93]	0.92 [0.87, 0.96]
Khartabil et al 2022	13	1	2	96	0.87 [0.60, 0.98]	0.99 [0.94, 1.00]
Picot et al 2022	109	4	0	244	1.00 [0.97, 1.00]	0.98 [0.96, 1.00]
Pillay et al 7a 2019	124	0	0	67	1.00 [0.97, 1.00]	1.00 [0.95, 1.00]
Pillay et al 7b 2019	272	1	6	749	0.98 [0.95, 0.99]	1.00 [0.99, 1.00]
Post et al 2019	253	2	104	478	0.71 [0.66, 0.76]	1.00 [0.99, 1.00]
Sarita et al 2011	52	35	18	325	0.74 [0.62, 0.84]	0.90 [0.87, 0.93]
Sharma et al. 2013	147	129	1	1974	0.99 [0.96, 1.00]	0.94 [0.93, 0.95]
Sunilkumar et al 2016	40	24	9	867	0.82 [0.68, 0.91]	0.97 [0.96, 0.98]
Yasuda et al 2022	32	0	1	47	0.97 [0.84, 1.00]	1.00 [0.92, 1.00]
Yoo et al 2010	191	4	222	1384	0.46 [0.41, 0.51]	1.00 [0.99, 1.00]
Zuluaga et al 2021	513	2	57	1154	0.90 [0.87, 0.92]	1.00 [0.99, 1.00]

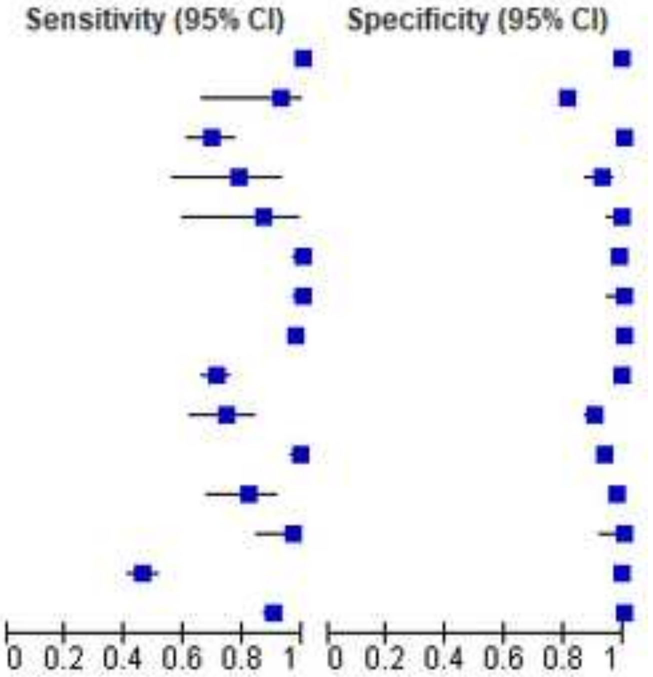

Click here to access/download;Figure;Fig.4..tif

sysmex with PCR

Study	TP	FP	FN	TN	Sensitivity (95% CI)	Specificity (95% CI)
Baya et al 2021	513	41	0	4477	1.00 [0.99, 1.00]	0.99 [0.99, 0.99]
Kagaya et al 2022	18	11	5	135	0.78 [0.56, 0.93]	0.92 [0.87, 0.96]
Picot et al 2022	109	4	0	244	1.00 [0.97, 1.00]	0.98 [0.96, 1.00]
Pillay et al 7a 2019	124	0	0	67	1.00 [0.97, 1.00]	1.00 [0.95, 1.00]
Pillay et al 7b 2019	272	1	6	749	0.98 [0.95, 0.99]	1.00 [0.99, 1.00]
Post et al 2019	253	2	104	478	0.71 [0.66, 0.76]	1.00 [0.99, 1.00]
Yasuda et al 2022	32	0	1	47	0.97 [0.84, 1.00]	1.00 [0.92, 1.00]
Yoo et al 2010	191	4	222	1384	0.46 [0.41, 0.51]	1.00 [0.99, 1.00]
Zuluaga et al 2021	513	2	57	1154	0.90 [0.87, 0.92]	1.00 [0.99, 1.00]

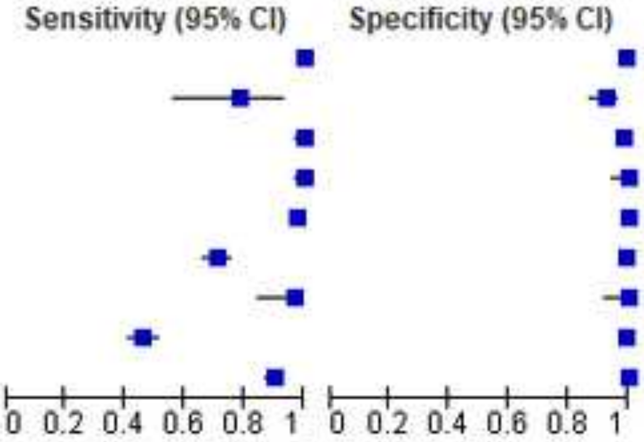

sysmex with microscope

Study	TP	FP	FN	TN	Sensitivity (95% CI)	Specificity (95% CI)
Buoro et al 2018	13	200	1	847	0.93 [0.66, 1.00]	0.81 [0.78, 0.83]
Hee et al 2008	100	0	44	343	0.69 [0.61, 0.77]	1.00 [0.99, 1.00]
Khartabil et al 2022	13	1	2	96	0.87 [0.60, 0.98]	0.99 [0.94, 1.00]
Sarita et al 2011	52	35	18	325	0.74 [0.62, 0.84]	0.90 [0.87, 0.93]
Sharma et al. 2013	147	129	1	1974	0.99 [0.96, 1.00]	0.94 [0.93, 0.95]
Sunilkumar et al 2016	40	24	9	867	0.82 [0.68, 0.91]	0.97 [0.96, 0.98]

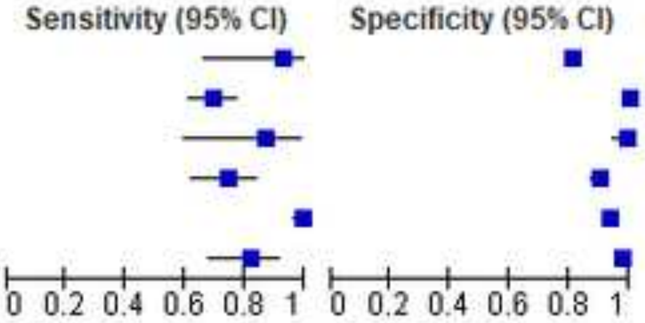

Click here to access/download;Figure;Fig.5..tif

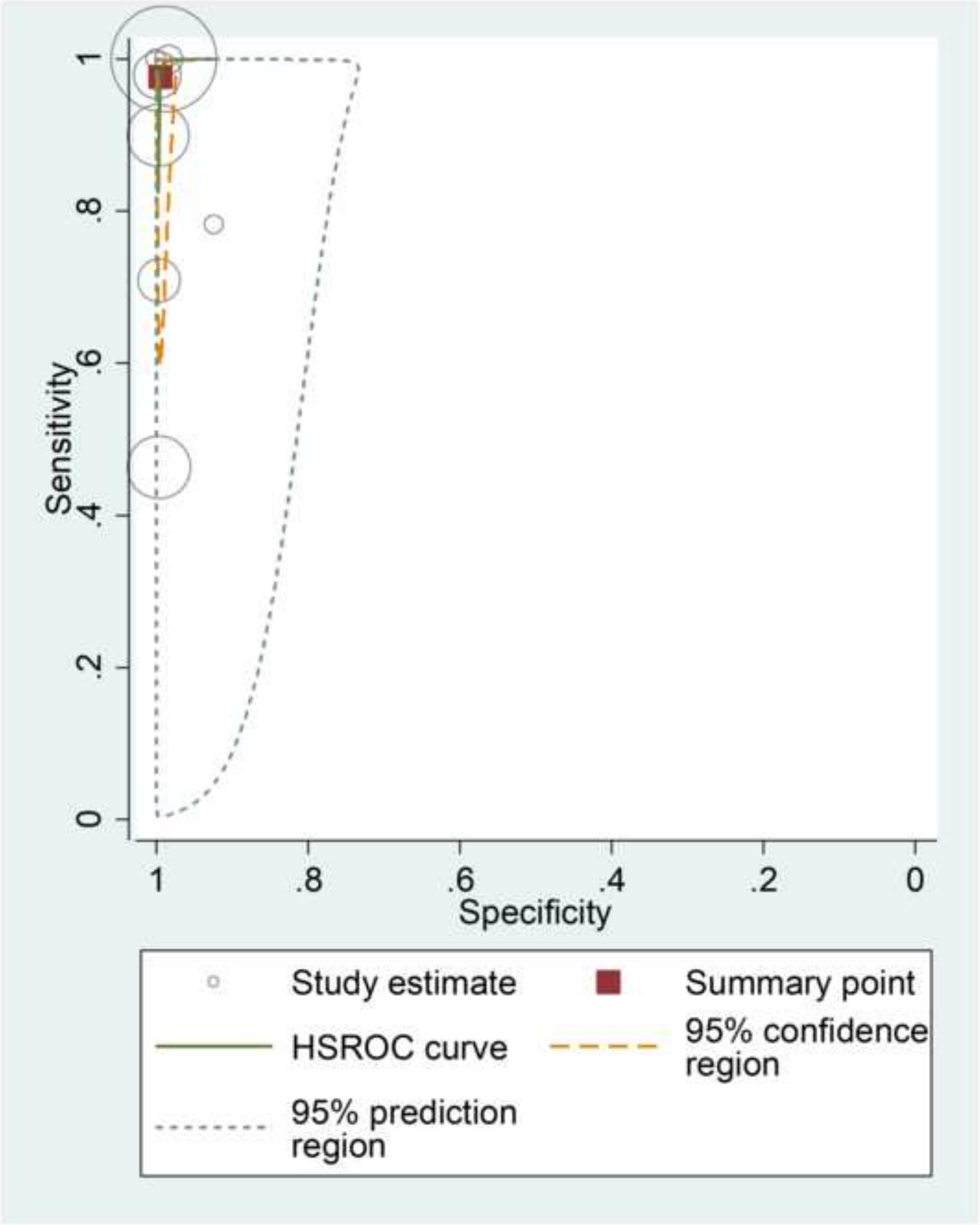

Click here to access/download;Figure;Fig.6..tif

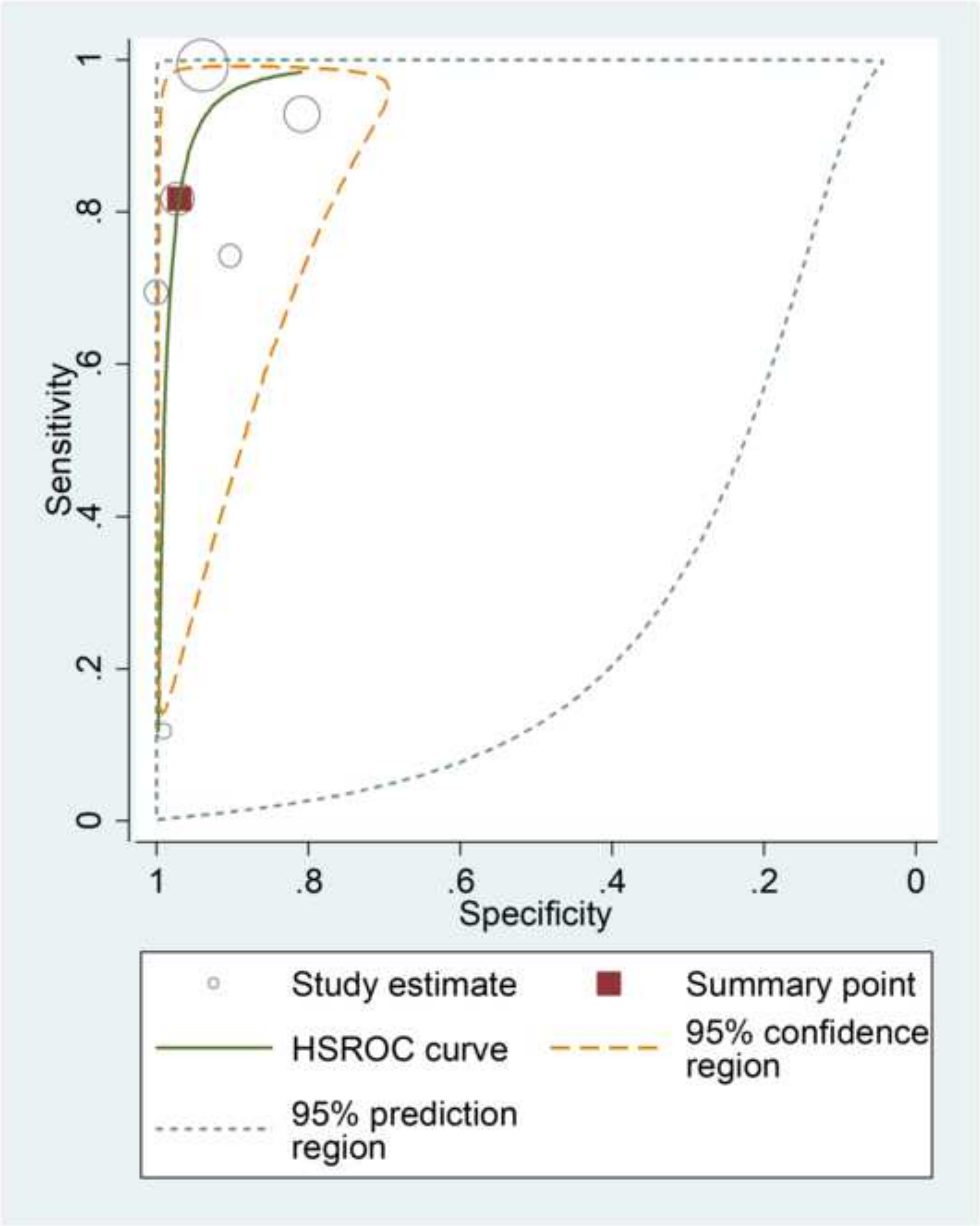

Click here to access/download;Figure;Fig.7..tif

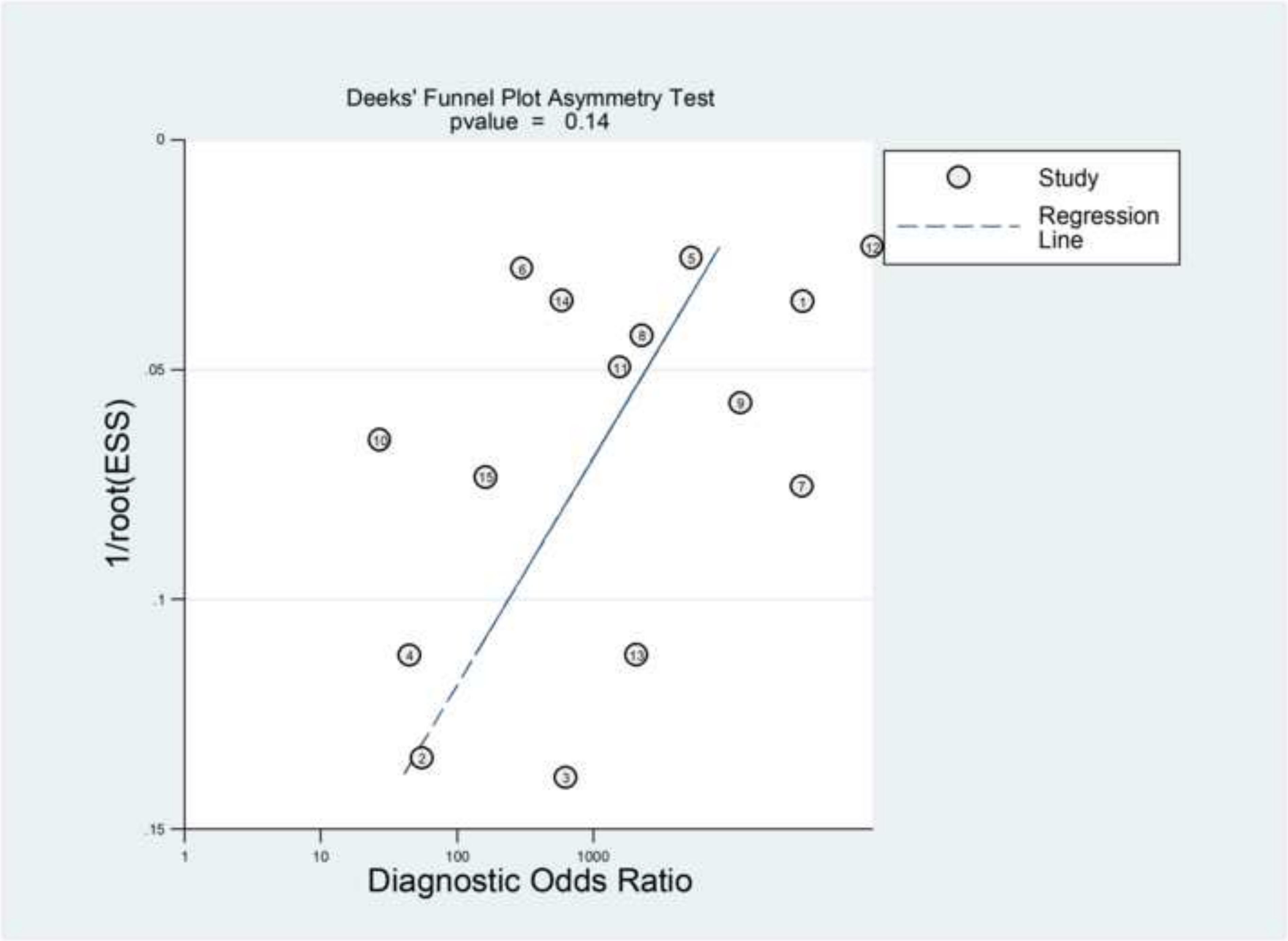

Click here to access/download
Supporting Information
S1.docx
