# Supplementary material for: Diagnostic role of Sysmex hematology analyzer in the detection of malaria: A systematic review and meta-analysis
Source: PLoS One. 2024 Sep 6;19(9):e0296766. doi: 10.1371/journal.pone.0296766 (PMC11379208; doi:10.1371/journal.pone.0296766)
Supplement: Attachment — Submitted filename: Response to Reviewers.docx [file pone.0296766.s003.docx]

**Author’s Response to editor and Reviewers**

**Diagnostic role of Sysmex hematology analyzer in the detection of malaria: A systematic review and meta-analysis.**

Zewudu Mulatie^1*^, Amanuel Kelem ^2^, Elias Chane ^3^, Amare Mekuanint Tarekegn ^3^, Bisrat Birke Teketelew^4^, Abebe Yenesew ^5^, Abatneh Melkamu ^5^, Yalew Muchie ^5^, Bedasa Addisu^2^, Dereje Mengesha Berta ^4^

**PLOSE ONE**

***Corresponding Author**: Zewudu Mulatie

Department of Medical Laboratory Sciences, College of Medicine and Health Science, Wollo University, Dessie, Ethiopia. P.O.Box 1145; email: [zewudumulatie@gmail.com](mailto:zewudumulatie@gmail.com) ; Tel: +251945274251

**Date:** Nov 21, 2023

**Dear PLOSE ONE** **Journal Editorial Office**

We would like to express our gratitude to the editorial teams and reviewers for their insightful comments and suggestions, which helped to make our manuscript, " **Diagnostic role of Sysmex hematology analyzer in the detection of malaria: A systematic review and meta-analysis**," more valuable and scientifically sound. The authors, tried to correct and include all the comments given by the reviewer point-by-point. For convenience purposes, we attached or uploaded the reply to the reviewer and the revised manuscript with track changes. We confirm that this manuscript has not been published elsewhere and is not under consideration by another journal. All authors have approved the manuscript and agreed with its resubmission to the PLOSE ONE Journal. All the reviewer comment responses are presented below.

**With regards!**

Zewudu Mulatie

Corresponding author

**Response to editor’s**

We thank you very much for your constructive comments and suggestions for the improvement of this manuscript. We have corrected and amended the manuscript based on your comments and suggestions. Herewith, please find in blue, the point-by-point response for editor’s comments and suggestions.

**Editor comments**

**Comment 1:** *Can one load a blood sample to test for malaria only? Or one has to run a CBC to look for parasites alongside a CBC?*

***Response****:* We would like to thank you for your questions. The Sysmex automated hematology analyzer works the Laboratory profession run a CBC to look for parasites alongside a CBC*.*

**Comment 2: *“****What happens when a patient only wants malaria test alone, or if this is not possible, What’s the economic implication if one has to run a malaria test alongside a CBC for malaria patients?”*

***Response****:* We would like to thank you for your concern. The cost of to run a malaria test alongside a complete blood count (CBC) for malaria patients are comparable to that of the cost of CBC. Because, the Sysmex automated haematology analyzer is a new fow cytometry-based device developed to measure the number and the ratio of malaria-infected red blood cells (MI-RBC) with a CBC. It can provide results in about one minute and also can simultaneously provide information on the malaria parasite (Plasmodium) species. Additionally, in the fact the CBC test is the most requested test when the patients febrile and suspected diseases.

**Comment 3: “*At what level of care within the system would the equipment be suitable for optimal utilization*?”**

***Response****:* We would like to thank you for your concern. The system or the Sysmex automated hematology analyzer more suitable for all hospitals and health centers. Because, this health facilities have the ability to fulfill the Sysmex automated hematology analyzer. However, to apply this automated hematology analyzer in across rural settings it may be affected by different factors such as stable electricity supply in a temperature-controlled room, cost of the instrument and its initial set-up, and need for stable reagent supply. Also, this system applies in malaria endemic and non-malaria endemic area to diagnosis malaria in asymptomatic and asymptomatic patients.

**Comment 4: “***Despite the challenges faced by RDTs, they are easy to use and can be used by all cadres as POCs including nurses, Lab techs, etc hence increasing access to parasite-based diagnosis; based on this, What would be health cadre to use this technology for malaria diagnosis in terms qualification (scientists, Lab techs, nurses or other cadres trained to test for malaria) and how does this affect access to testing and affect the equipment advantage?”*
***Response****:* We would like to thank you for your concerns. This automated hematology analyzer requires minimal training and is easily accessible in most healthcare facilities. Notably, it can analyze each sample in approximately 1 minute, encompassing specimen aspiration, result generation, and measurement performance—all requiring limited technical input or expertise. Furthermore, it enables the accurate reporting of infected red blood cells and percentage parasitemia, facilitating the monitoring of parasite load reduction and therapeutic efficacy post-initiation of anti-malarial treatment. These properties may enable the early identification of drug resistance to the current frontline artemisinin-based combination therapy.

Additionally, in addition to its unique ability to directly identify parasites in red blood cells, it produces a conventional complete blood count (CBC) with each result. This concurrent detection of malaria parasites and generation of a CBC may be valuable in identifying previously unsuspected cases of malaria. A CBC is typically part of the initial diagnostic blood panel for febrile patients, and the automated analyzer will automatically flag the sample if malaria parasites are present. In contrast, other diagnostic tools necessitate a specific request from a healthcare professional to test for malaria.

Moreover, it serves as a screening tool for malaria in donors in blood banks situated in malaria-endemic regions. The simultaneous hematological evaluation may also offer insights into the donor's health status. Another significant benefit of the Sysmex automated hematology analyzer is its capability to detect stages of parasites, including the gametocyte stage. Furthermore, this analyzer rapidly identifies the actual parasite and not any by-products, such as antigens, phagocytized parasites, or haemozoin, rendering it more suitable for malaria detection when compared to RDTs and other indirect automated methods.

***Comment 5:*** *Were these mostly done in Africa where the malaria burden is, what was the distribution of the reviewed studies?*

***Response****:* We would like to thank the editor for your questions. The studies done in Africa were in Malawi, Kenya, Burkina Faso and 2 studies in South Africa. Most of studies done in Africa and Asia. This indicates the Sysmex hematology analyser used for malaria diagnosis in malaria endemic and non-malaria endemic area.

***Comment 6:*** *How many types or versions of sysmex equipment are able to do malaria testing? Did the studies cover all versions or one equipment version and what’s the implication?*
***Response****:* We would like to thank the editor for your questions. By our knowledge or extensive searching, there are 3 versions of sysmex hematology analyzer for malaria detection. Such as, XN, XT and XE. The studies cover only the 2 versions (XN and XE). But there is 1 case report about XT. The implication of studies showed that the XN versions have high sensitivity and specificity and more used for clinical utility.

***Comment 7:*** *What patient populations (symptomatic, asymptomatic, age, parasite densities, etc, could u provide a table summarizing patient baseline characteristics?*

***Response****:* We would like to thank you. We did as your suggestion

**Comment 8**: Line: 108: revise the sentience- uantity?

***Response****:* We would like to thank you. We did as your suggestion

**Comment 9**: *Line 132-33: How many non-English articles/studies were excluded? Unpublished reports and conference papers?*

***Response****:* We would like to thank the editor for your concerns. We considered excluding non-English articles, unpublished reports and conference reports. However, we did not get as such studies.

**Comment 10**: *Line 159: whats the authors comment on using a sample of 15 papers for a systematic review to draw the conclusions?*

***Response****:* We would like to thank the editor for your concerns. We extensively searched the studies and get 15 studies. So, this systematic review enough to draw conclusion

***Comment 11****: Line 165-67: what was the reference/gold standard?*

***Response****:* We would like to thank the editor for your concerns. The gold standard for 9 studies was polymerase chain reaction and for 6 studies the gold standard/reference was microscope.

**Response to reviewer(s’) comments**

On behave of the authors, I would like to thank reviewers for their constructive and scholarly comments that are valuable to make the paper scientifically sound. The comments are well accepted and most of the suggestions made by the reviewers were incorporated. Those changes are highlighted in blue within the manuscript. Herewith, please find in blue, the point-by-point response for reviewers’ comments and suggestions.

**REVIEWER #1 General comments**

**Comment 1: “***The study's objective is to assess Sysmex's diagnostic performance in diagnosing malaria; however, the cost was not considered by the authors. Cost is quite important, particularly in areas where malaria is a major problem*.”

***Response****:* We would like to thank you for your concern. The cost of sysmex to diagnosis of malaria is comparable to that of the cost of CBC. Because, the Sysmex automated haematology analyzer is a new flow cytometry-based device developed to measure the number and the ratio of malaria-infected red blood cells (MI-RBC) with a CBC. It can provide results in about one minute (it saving time) and also can simultaneously provide information on the malaria parasite (Plasmodium) species. Additionally, in the fact the CBC test is the most requested test when the patients febrile and suspected diseases. Also, have many advantages compared to other methods (RDT).

***Comment 2:*** *Where this device will be used was not specified by the authors. Is it in the comprehensive hospital, primary hospital, or health center? Can this device be used in health post-level settings to replace the existing RDT?*

***Response****:* We would like to thank you for your concern. The sysmex authomated hematology analyzer most found in comprehensive hospital, primary hospital and health center. In the current situation, the sysmex automated hematology analyzer difficult to replace the existing RDT. But sysmex automated machine have advantage other than RDT, such as it quantify parasite, has short TAT, it detect parasite stage, to detect parasite at low concentration, for identifying the health status of the patient and used treatment monitoring by using parasite clearance.

***Comment 3:*** *The authors advised to revise the conclusion section. The conclusion fails to persuade the readers based on the findings.*

***Response****:* We would like to thank the reviewer for the comments. We did as you suggestion.

***Comment 4:*** *Apart from PCR, the diagnostic performance of LAMP is extensively studied and recommended by most scientists in the fields. Nevertheless, the authors didn't mentioned about LAMP in the background section.*

***Response****:* We would like to thank the reviewer for the comments. We did as you suggestion.

***Comment 5:***	*Control and eradication were terms employed by the authors in the body of the manuscript. They overlooked a critical step "elimination". The elimination process is crucial to the activities of a particular country or subregional program. For instance, in Ethiopia (where the current program is malaria elimination) or other East African countries where gene deletions are currently highly prevalent, this method may be suggested for the diagnosis of malaria.*

**Response**: We would like to thank the reviewer for the comments. We did as you suggestion.

***Comment 6:*** *The authors advised italicizing every malaria species mentioned in the text's main body.*

**Response**: We would like to thank the reviewer for the comments. We did as you suggestion.

***Comment 7:*** *In the background the cost implication of using PCR was not mentioned.*

**Response**: We would like to thank the reviewer for the comments. We already did as stated.

***Comment 8:*** *What more advantages come with using a Sysmex hematological analyzer than those already mentioned?*

***Response****:* We would like to appreciate your concerns. The automated hematology analyzer requires minimal training, is accessible in most health facilities, and is simpler to use compared to molecular methods. It boasts several advantages: it can analyze each sample in approximately 1 minute, including specimen aspiration, measurement performance, and result reporting, requiring minimal technical expertise compared to molecular and thin blood film methods. Additionally, it provides an accurate count of infected red blood cells and percentage of parasitemia, enabling the monitoring of parasite load reduction and therapeutic efficacy following the initiation of anti-malarial treatment. These properties may facilitate early identification of drug resistance to the current frontline artemisinin-based combination therapy, unlike RDT.

Another significant advantage is its unique ability to directly identify parasites in red blood cells while generating a conventional complete blood count (CBC), providing valuable insights into previously unsuspected cases of malaria—something other malaria diagnostic tools cannot accomplish. A CBC is commonly part of the initial diagnostic blood panel for febrile patients, and the automated analyzer will automatically flag the sample if malaria parasites are detected. In contrast, all other diagnostic tools require a specific request from a healthcare professional to test for malaria. Furthermore, it serves as a malaria screening tool for donors in blood banks located in malaria-endemic regions. The simultaneous hematological evaluation may also offer insights into the donor's health status.

Lastly, the Sysmex automated hematology analyzer's ability to detect parasite stages, including the gametocyte stage, sets it apart from RDT. Moreover, this analyzer rapidly identifies actual parasites without capturing by-products such as antigens, phagocytized parasites, or haemozoin, making it more suitable for malaria detection compared to RDTs and other indirect automated methods.

***Comment 9:*** *The year of studies indicated in the abstract and the paper body are different, so please correct them*.

***Response****:* We would like to thank you for your concerns. We did as suggested

***Comment 10:*** Table-1 need revisions. It would be nice to include one column for reference
***Response****:* We would like to thank you for your concerns. We did as suggested

Specific comments:
***Comment 11:*** -Line 176, in the nine studies are ?? needs revision.

***Response****:* We would like to thank you for your comments. We did as suggested
***Comment 12:*** Line 276. "Because of has high diagnostic accuracy" needs revision.

***Response****:* We would like to thank you for your comments. We did as suggested
***Comment 13:*** Table 2 needs formatting.

***Response****:* We would like to thank you for your comments. We did as suggested
***Comment 14:*** The conclusion must be changed in line with the findings.

***Response****:* We would like to thank you for your comments. We did as suggested

**REVIEWER #2 General comments**

Please revise grammar and spelling errors, I am listing some here. I have also suggested some options to paraphrase some sentences for clarity.

***Response****:* We would like to thank you for your comments. We did as suggested

• 108: uantity =quantity

***Response****:* We would like to thank you for your comments. We did as suggested

• 214: significant, confidence

***Response****:* We would like to thank you for your comments. We did as suggested
• 237: Sysmex

***Response****:* We would like to thank you for your comments. We did as suggested
• 253: malaria-related complications

***Response****:* We would like to thank you for your comments. We did as suggested
• 254: into the routine

***Response****:* We would like to thank you for your comments. We did as suggested
• 252: plasmodium=Plasmodium

***Response****:* We would like to thank you for your comments. We did as suggested
• 255: instead of “negative consequences…” consider “complications of malaria”.

***Response****:* We would like to thank you for your comments. We did as suggested
• 256: not requires = does not require

***Response****:* We would like to thank you for your comments. We did as suggested

• 264: associated with

***Response****:* We would like to thank you for your comments. We did as suggested

• 265: consider replacing “ the patient, parasitaemia “ with “ the level of parasitaemia”.

***Response****:* We would like to thank you for your comments. We did as suggested
• 266-267: consider replacing “for Sysmex hematology analyzer” with “for the Sysmex hematology analyzer ..”

***Response****:* We would like to thank you for your comments. We did as suggested
• 269: estimate sensitivity =estimated sensitivity

***Response****:* We would like to thank you for your comments. We did as suggested

• 272-273: obtaining a positive result in a diseased person than in a non-diseased one

***Response****:* We would like to thank you for your comments. We did as suggested
• 274: a positive individual and a negative one

***Response****:* We would like to thank you for your comments. We did as suggested
• 276:” diagnosis. Because” = ” diagnosis because”

***Response****:* We would like to thank you for your comments. We did as suggested
• 282-283: “diagnostic accuracy ….” This sentence is not clear , consider replacing it with “ the microscopic diagnosis of malaria tends to be less sensitive in low parasitaemia and has been reported as 70% in pregnancy-associated malaria [36].

***Response****:* We would like to thank you for your comments. We did as suggested
• 287-288: “which stated a sensitivity…” consider “ which reported the sensitivity of PCR to be 98%”

***Response****:* We would like to thank you for your comments. We did as suggested
• 291: ….the gold standard was shown to have a higher ….

***Response****:* We would like to thank you for your comments. We did as suggested

**REVIEWER #3 General comments**

The manuscript followed sound methodological and statistical strategies, but has some typological and grammatical errors that need to be corrected to make it clear and easy to read. The discussion did not include the "and so what" of their findings and the implications of the findings.

***Response****:* We would like to thank you for your comments. We tried to corrected and incorporated your concern

**Response to the attached file**

What is Ste and S?

***Response****:* We would like to thank you for your comments. They are the constant term during meta-regression analysis.

How does the Sysmex analyzer integrated malaria treatment?

***Response****:* We would like to thank you for your comments. Because it used for count parasite level or parasite clearance, by identifying stages of malaria parasite, to assess the health status of the patient by using CBC and it identify parasite early in the un suspected malaria patients (asymptomatic patients)

Other comments and suggestions raised by reviewer

***Response****:* We tried to corrected and incorporated in the manuscript.
